# Very long chain fatty acids are an important marker of nutritional status in patients with anorexia nervosa: a case control study

**DOI:** 10.1186/s13030-020-00186-8

**Published:** 2020-07-17

**Authors:** Miki Shimizu, Keisuke Kawai, Makoto Yamashita, Masayasu Shoji, Shu Takakura, Tomokazu Hata, Megumi Nakashima, Keita Tatsushima, Kazunari Tanaka, Nobuyuki Sudo

**Affiliations:** 1 Department of Psychosomatic Medicine, Graduate School of Medical Science, Kyushu University, 3-1-1 Higashi-ku, Fukuoka, 812-8582 Japan; 2 Department of Psychosomatic Medicine, Kohnodai Hospital, National Center for Global Health Medicine, 1-7-1, Kohnodai, Ichikawa City, Chiba 272-8516 Japan; 3 Department of Nutrition, University of Nagasaki, 1-1-1 Manabino, Nagayo-cho, Nishisonogi-gun, Nagasaki, 851-2195 Japan

**Keywords:** Anorexia nervosa, AN-restricting type, AN-binge-eating/purging type, Fatty acids, Very long chain fatty acids, Lipid metabolism

## Abstract

**Background:**

Anorexia nervosa (AN) is a disease resulting in extreme weight loss. It is caused by multiple factors, including psychosocial, environmental, and genetic factors. A genetic abnormality affecting lipid metabolism has been recently reported in patients with AN. However, it is unknown whether lipid metabolism abnormalities in AN are caused by eating behavior, undernutrition, and/or genetic factors. The meaning of lipid metabolism in AN remains unclear. In particular, differences in the profiles of very long-chain fatty acids (VLCFAs) in patients with various types of AN have not been studied. This study aimed to determine changes to the fatty acid profile over a 3-month period, specifically that of long-chain fatty acids (LCFAs) and VLCFAs in patients with various types of AN.

**Methods:**

We evaluated 69 female patients with AN, subclassified as AN-restricting type (AN-R) and AN-Binge-Eating/Purging type (AN-BP). On admission and after 3 months of treatment, height, weight, body mass index, plasma and serum parameters, and plasma fatty acid concentrations were measured in all patients. The control group included 25 healthy, age-matched women. Comparisons between the groups were made using one-way ANOVA, while those between the various parameters at admission and after 3 months within each group were made using the Wilcoxon signed rank test.

**Results:**

On admission, the AN-R and the AN-BP groups had significantly higher levels of 18-24C and > 14C fatty acids (LCFAs and VLCFAs, respectively) than the control group. After 3 months of treatment, both groups showed high levels of 14-24C fatty acids. The levels of VLCFAs (C22:0 and C24:0) and LCFA (C18:3) after 3 months of treatment remained high in both AN groups relative to the control group.

**Conclusions:**

Eating behaviors appear to be associated with levels of LCFAs. Lipid metabolism abnormalities under conditions of starvation in AN might have a genetic basis and appear to be associated with VLCFA (C22:0 and C24:0) and LCFA (C18:3) levels.

## Background

Anorexia nervosa (AN) is a disease that arises from an intense fear of gaining weight and results in extreme weight loss. It is caused by multiple factors, including psychosocial environmental, and genetic factors [1–3]. In AN, undernutrition can result in a life-threatening condition [3, 4]. This condition is often complicated by electrolyte abnormalities and severe sarcopenia, as well as hematological disorders, cardiac disturbances, and liver function abnormalities [4–9].

In 2019, a genome-wide association study based on data from the Wellcome Trust Case Control Consortium (WTCCC)-3, and the United Kingdom Biobank reported genetic associations between AN and mental disorders such as obsessive-compulsive disorder, schizophrenia, and major depressive disorder, as well as metabolic factors such as body mass index (BMI), obesity, fasting insulin levels, insulin resistance, and leptin production (a negative association) [10]. Shih et al. and Scott-Van Zeeland reported that epoxide hydrolase 2 activity was associated with fat metabolism in a susceptibility gene study [11, 12]. These findings have caused a paradigm shift in the pathologic understanding of AN. In fact, genetic/genomic studies have provided amble evidence that abnormalities in lipid metabolism may be an important factor underlying AN pathophysiology.

Lipid metabolism in AN is relatively understudied compared to other AN-related physical abnormalities such as severe sarcopenia, electrolyte abnormalities, hematological disorders, cardiac disturbances, and liver dysfunction. A previous study on the plasma fatty acid concentrations in patients with AN has shown that the concentrations of essential fatty acids were within the normal range, whereas those of polyunsaturated fatty acids were reduced [13]. Moreover, a selective decrease was observed in the levels of essential fatty acids, with a concurrent compensatory increase in the concentrations of short-chain saturated fatty acids and monounsaturated fatty acids [14–16]. However, overall the findings have been inconsistent [17], and no studies have evaluated the differences in lipid metabolism in patients with different types of AN. In the case of children with chronic malnutrition, such as marasmus, previous studies have reported on liver function abnormalities due to a decrease in the number of normal peroxisomes, disorders related to beta-oxidation, and the accumulation of very long chain fatty acids (VLCFAs) [18]. Zak et al. reported that increased absorption of exogenous cholesterol influenced the change of plasma lipids in AN [19].

We hypothesized that lipid metabolism abnormalities in AN are influenced by eating behavior, genetic factors, and undernutrition. This study aimed to determine changes to the fatty acid profile over a 3-month period, specifically that of long chain fatty acids (LCFAs) and VLCFAs in patients with various types of AN.

## Methods

Case subjects included 69 female patients with AN admitted for more than 3 months to the Department of Psychosomatic Medicine at the Kyushu University Hospital between 2011 and 2013. The diagnosis of AN was based on the guidelines described in the eating disorders section of the Structured Clinical Interview for DSM-IV Axis I Disorders [2]. Of the patients, 38 had the AN-restricting type (AN-R) and 31 had the AN-Binge-Eating/Purging type (AN-BP). Of the AN-BP patients, 27 resorted to self-induced vomiting, three misused laxatives or diuretics, and nine did both. The exclusion criteria for the study were severe mental illness, acute drug toxicosis, and severe liver damage during inpatient treatment. Patients who continued purging behavior during hospitalization were also excluded from the study. The control subjects were 25 healthy women who (a) were of normal weight, (b) had no dietary restrictions, (c) were not taking any medications, and (d) did not exhibit any abnormal eating behaviors. There was no significant age difference between the patients and controls.

### Treatment of patients with AN

An important treatment for AN is cognitive-behavioral therapy [20, 21]. The behavioral observation was performed during the first 2 or 3 weeks of admission. During this period, electrolyte abnormalities, including dehydration and edema, were almost normalized. After the behavioral observation, the treatment program was started and continued until a patient achieved the target body weight. In principle, the following conditions were maintained: behavioral limitations, regular personal interviews scheduled twice a week, and complete consumption of meals. In the initial stage of the treatment program, the patients were requested to remain in their rooms. As body weight increased, the limitations on behavior were removed, based on our behavioral protocol governing privileges. A patient who could not ingest the minimum nourishment of approximately 35 kcal per 1 kg in body weight received nasogastric tube feeding, after providing consent, to compensate for the lack of oral feeding. After the therapists confirmed that the patient could consume an entire meal without difficulty, the amount of each meal was increased gradually by approximately 200 kcal, and the feeding via the nasogastric tube was slowly reduced. The weight target was an increase of 500 g or more in 1 week. Therefore, the therapist gradually increased the total calorie intake of the patients. The daily energy intake was increased gradually to a maximum of 2000 to 2400 kcal/day. Purging behaviors were restricted as much as possible. Energy control foods offered at our hospital have a 23–28% fat content equivalent to energy intake of 1200–1600 kcal/day, which is lower than that in equivalent foods found in Western diets, in particular those offered in the early stages of AN treatment. Ensure liquid® supplementation was done. One month after starting treatment, the fat content of the offered meals was the same as that of Western meals.

### Measurement of parameters

On admission and after 3 months of treatment, body mass (dual-energy X-ray absorptiometry) and plasma fatty acid concentrations (gas chromatography) were measured in all patients. The blood for testing was drawn early in the morning (7:00 AM) after overnight fasting (from 10:00 PM of the day before) on the day after hospitalization and again 3 months later. However, the following were allowed: infusion of extracellular fluid (500 ml/12 h) or uptake of distilled water at nighttime. After centrifuging the blood sample, the serum and plasma were cryopreserved at − 80 °C until further testing. Fatty acid concentration and glucose levels were measured in the plasma; other biological parameters were measured in the serum. After saponification and washing with organic stratum methyl ester, the fatty acid concentration was measured by gas chromatography [13]. The biochemical parameters of the serum and plasma glucose were measured in the hospital laboratory, and dual-energy X-ray absorptiometry was performed within 1 week of blood sampling for the measurement of all variables of interest in the present study.

Fatty acids were divided into saturated fatty acids, i.e., without double bonds between carbon atoms, monounsaturated fatty acids (MUFAs: n-7, n-9) with one double bond, and polyunsaturated fatty acids (PUFAs: n-6, n-3) with two or more double bonds. Short-chain fatty acids contain < 6 carbon atoms, medium-chain fatty acids contain 8–10 carbon atoms, long-chain fatty acids (LCFAs) contain 12–20 carbon atoms, and VLCFAs contain > 20 carbon atoms. LCFAs are related to energy metabolic function and cell adhesion, and VLCFAs are related to the biochemistry of adipose tissue, generative function, and aging.

### Statistical analysis

One-way ANOVA and the Dunnett test were used for comparisons among groups. The Wilcoxon signed rank test was used to compare differences between various measured parameters at admission and after 3 months within each group. All analyses were performed using the JMP® 11 (SAS Institute Inc., Cary, NC, USA) software and the results are presented as mean ± standard deviation (SD). The criterion for statistical significance was a *P*-value < 0.05.

### Ethical considerations

All participants provided written informed consent at the time of admission, and the study protocol was approved by the Kyushu University Research and Ethics Committee [15–25].

## Results

### Physical characteristics

Table 1 summarizes the physical characteristics of the subjects at the time of admission and after 3 months of treatment. On admission, the weight and BMI of patients in the AN-R and AN-BP groups were significantly lower than those of the control individuals (*P* < 0.05). No significant difference in body composition was found between the groups. At the end of 3 months of treatment, the body weight of those in the AN-R and AN-BP groups had increased significantly, but they had still not reached the weight of the control group.

**Table 1:** Demographic data at admission and after 3 months of treatment

	AN-R (*n* = 38)		AN-BP (*n* = 31)		Control group
	on admission	after 3 months	on admission	after 3 months	(*n* = 25)
Age (years)	28.42 ± 12.39	28.42 ± 12.39	32.43 ± 8.19	32.30 ± 8.19	29.33 ± 6.55
Duration of anorexia (days)	2114.08 ± 3255.41	NR	2629.29 ± 2037.48	NR	NR
Height (cm)	154.44 ± 5.39∗	154.77 ± 5.85∗	158.27 ± 3.59	158.47 ± 3.71	160.16 ± 3.02
Body weight (kg)	30.73 ± 5.50∗	37.86 ± 6.04∗♱	30.33 ± 3.76∗	38.12 ± 5.03∗♱	51.99 ± 4.79
Body mass index (kg/m^2^)	12.81 ± 2.00∗	15.77 ± 2.15∗♱	12.17 ± 1.58∗	15.25 ± 2.00∗♱	20.20 ± 1.91
Fat mass (g)	3498.3 ± 1611.31	8272.79 ± 3604.42♱	4054.34 ± 1572.67	9557.05 ± 5310.85♱	NR
Lean body mass (g)	27,283.02 ± 3242.18	30,321.15 ± 3556.30♱	26,914.63 ± 2493.5	26,616.08 ± 2334.50♱	NR
Percentage of fat mass (%)	10.62 ± 3.81	20.04 ± 7.09♱	12.07 ± 3.70	20.42 ± 3.77♱	NR

*AN-R* Anorexia Nervosa Restricting type, *AN-BP* Anorexia Nervosa Binge-Eating/Purging type

NR: denotes data not reported. Values presented as the mean ± SD for the number of observations indicated

Significant differences in the comparisons of AN-R and AN-BP with controls: ∗*P* < 0.05

Significant differences between values on admission and after 3 months: ♱*P* < 0.05

### Serum- and plasma-based parameters

Table 2 summarizes the serum and plasma parameters at the time of admission and 3 months later. On admission, the mean triglyceride (TG) level of the AN-BP group was significantly higher than that of the control group (*P* < 0.05). The mean free triiodothyronine (FT3) levels of both the AN-R and AN-BP groups were significantly lower than those of the control group (*P* < 0.05). The mean free thyroxine (FT4) level of the AN-R group was significantly lower than that of the control group (*P* < 0.05). The mean cortisol levels of the AN-R and AN-BP groups were significantly higher than those of the control group (*P* < 0.05).

**Table 2:** Biochemical parameters on admission and after 3 months of treatment

	AN-R (*n* = 38)		AN-BP (*n* = 31)		Control group
	on admission	after 3 months	on admission	after 3 months	(*n* = 25)
Glucose (mg/l)	79.79 ± 16.93	80.32 ± 7.73	76.7 ± 12.09∗	81.42 ± 7.42♱	85.40 ± 8.95
Total cholesterol (mg/dl)	189.32 ± 60.08	193 ± 37.17	228.83 ± 50.95∗	208.68 ± 42.71	200.16 ± 28.46
Triacylglycerol (mg/dl)	74.24 ± 37.28	73.55 ± 23.06	108.73 ± 47.99∗	83.26 ± 44.51	58.54 ± 19.45
HDL-C (mg/dl)	70.47 ± 19.63	69.91 ± 17.46	74.2 ± 25.32	70.79 ± 15.66	74.24 ± 16.42
TSH (μU/ml)	2.61 ± 2.27	2.31 ± 1.29	1.629 ± 1.16	1.79 ± 0.65	2.01 ± 0.72
FT3 (pg/ml)	1.93 ± 0.60∗	2.71 ± 0.69∗♱	2.06 ± 0.53∗	2.35 ± 0.32∗♱	3.21 ± 0.40
FT4 (ng/dl)	1.07 ± 0.24∗	0.92 ± 0.18∗♱	1.16 ± 0.33	0.92 ± 0.18∗♱	1.29 ± 0.20
Cortisol (μg/dl)	20.19 ± 6.56∗	12.79 ± 4.11♱	20.33 ± 6.47∗	13.26 ± 4.09♱	15.68 ± 3.70
GH (nU/ml)	12.30 ± 19.84∗	3.01 ± 6.78♱	8.65 ± 14.00	2.52 ± 1.94♱	2.50 ± 2.67
AST (IU/l)	43.82 ± 48.32	34.81 ± 23.30	72.48 ± 203.63	34.83 ± 27.36	NR
ALT (IU/l)	50.34 ± 40.26	47.36 ± 39.67	53.00 ± 99.29	42.17 ± 26.80	NR

*AN-R* Anorexia Nervosa Restricting type, *AN-BP* Anorexia Nervosa Binge-Eating/Purging type

FT3: free triiodothyronine, FT4: free thyroxine, GH: growth hormone

Values presented as the mean ± SD for the number of observations indicated

Significant differences in the comparisons of the AN-R and AN-BP groups with controls: ∗*P* < 0.05

Significant differences between values on admission and after 3 months: ♱*P* < 0.05

NR: denotes data not reported. The normal ranges for AST and ALT are 7–38 IU/L and 4–44 IU/L, respectively, based on the reference range used by our laboratory

After 3 months of treatment, the serum- and plasma-based parameter levels were closer to normal values. The mean FT3 levels of the AN-R and AN-BP groups were significantly lower than that of the controls.

The mean aspartate aminotransferase (AST) and alanine aminotransferase (ALT) values in the AN-R and AN-BP group at the time of admission were slightly in excess of the normal range indicated for the Japanese population (Table 2). However, after 3 months of treatment these values decreased in both groups, although the decrease was not statistically significant (Table 2).

### Plasma fatty acid profiles

**Fig. 1:**
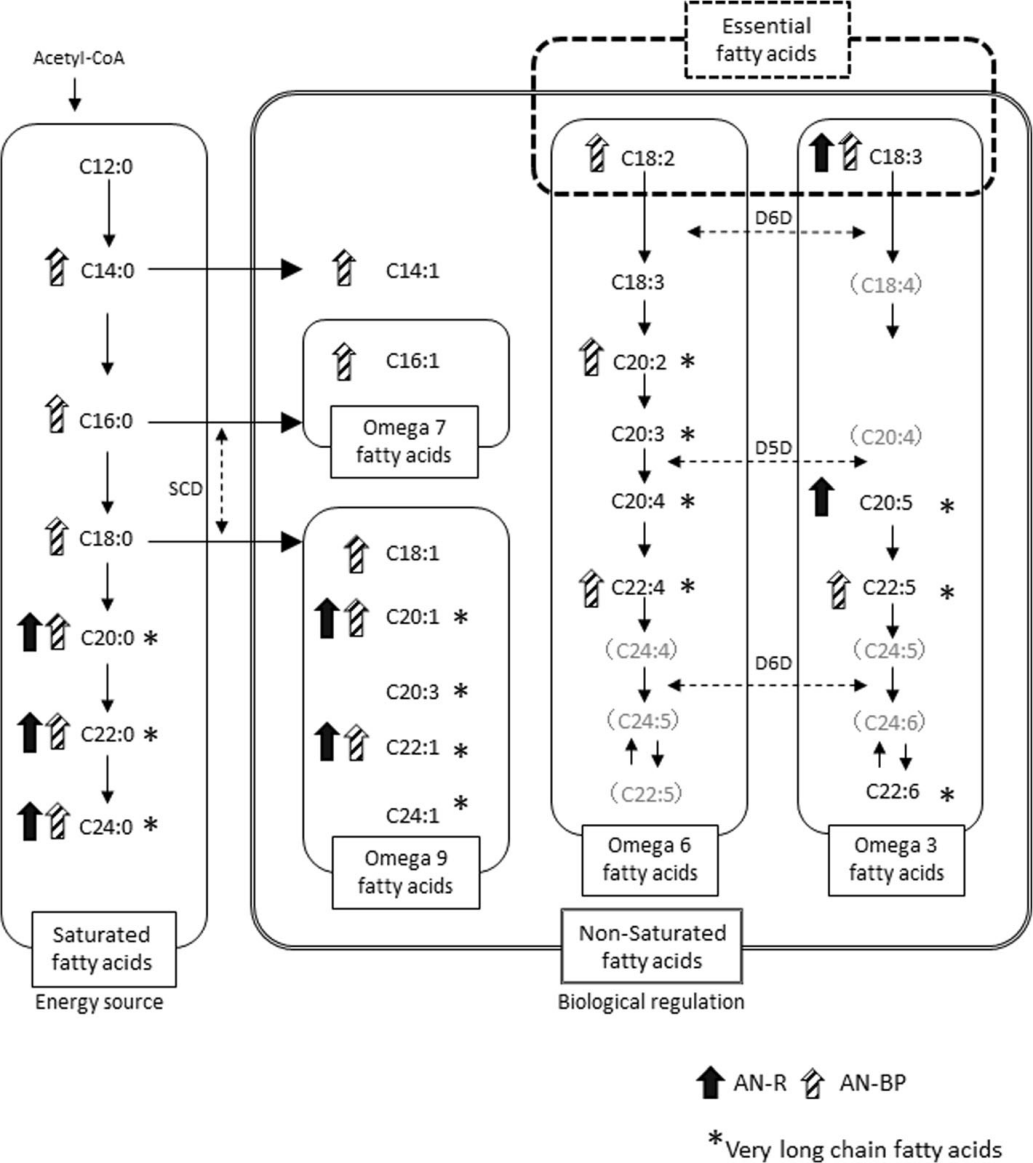
Metabolism of fatty acids in the AN-R and AN-BP groups on admission. AN-R: Anorexia Nervosa restricting type, AN-BP: Anorexia Nervosa Binge-Eating/Purging type, SCD: stearoyl-CoA desaturase, Δ6D: delta-6 desaturase, Δ5D: delta-5 desaturase. The black and the oblique line arrows show the fatty acid levels in the AN-R and AN-BP groups, respectively. The arrows indicate fatty acid levels that are significantly higher in comparison with those of the healthy controls. Significance level of the difference in comparison with the controls: *P* < 0.05

Table 3 shows the plasma fatty acid profiles on admission and after 3 months of treatment. In the AN-R group on admission, the levels of 18C or longer fatty acids, saturated fatty acids, and MUFAs (VLCFAs) and PUFAs (VLCFA) were significantly higher than those in the control group (Fig. 1). In contrast, the AN-BP group had high levels of a range of saturated fatty acids, MUFAs, and PUFAs of 14-24C (LCFAs+VLCFAs) (Fig. 1).

**Table 3:** Fatty acid composition on admission and after 3 months of treatment

	AN-R(*n* = 38)		AN-BP (*n* = 31)		Control group
	on admission	after 3 months	on admission	after 3 months	(*n* = 25)
C12:0 (μg/ml)	2.62 ± 1.98	3.10 ± 1.41	3.82 ± 3.17	3.84 ± 3.19	3.66 ± 4.16
C14:0 (μg/ml)	16.12 ± 5.59	25.58 ± 7.04∗♱	21.78 ± 8.85∗	29.89 ± 13.75∗	15.48 ± 6.20
C14:1n-5 (μg/ml)	1.42 ± 0.71	2.15 ± 0.87∗♱	2.27 ± 1.27∗	3.01 ± 1.58∗	1.29 ± 0.65
C16:0 (μg/ml)	488.11 ± 162.94	493.89 ± 93.78	603.93 ± 156.37∗	537.83 ± 130.01	457.96 ± 70.72
C16:1n-7 (μg/ml)	42.55 ± 23.35	46.47 ± 19.09	83.35 ± 44.26∗	55.39 ± 35.42♱	35.64 ± 11.76
C18:0 (μg/ml)	231.11 ± 49.62	243.47 ± 39.59	274.06 ± 63.23∗	263.44 ± 41.13	232.80 ± 39.22
C18:1n-9 (μg/ml)	523.11 ± 253.63	406.00 ± 68.07	721.10 ± 209.79∗	458.22 ± 126.84♱	461.12 ± 100.02
C18:2n-6 (μg/ml)	794.34 ± 217.25	736.31 ± 170.57	973.61 ± 286.45∗	851.22 ± 173.29	820.48 ± 160.14
C18:3n-6 (μg/ml)	5.71 ± 4.90	8.71 ± 5.44	8.40 ± 5.67	9.94 ± 5.17∗	6.11 ± 3.32
C18:3n-3 (μg/ml)	20.48 ± 8.39∗	22.95 ± 7.01∗	24.86 ± 8.53∗	25.78 ± 10.44∗	15.72 ± 5.57
C20:0 (μg/ml)	2.04 ± 0.57∗	1.83 ± 0.41	2.15 ± 0.66∗	1.82 ± 0.39	1.56 ± 0.29
C20:1n-9 (μg/ml)	6.07 ± 2.40∗	5.54 ± 2.64	5.89 ± 2.19∗	6.57 ± 3.5∗	4.26 ± 0.86
C20:2n-6 (μg/ml)	5.71 ± 3.25	5.83 ± 1.39∗	6.87 ± 3.78∗	7.26 ± 2.01∗	4.69 ± 1.26
C20:3n-9 (μg/ml)	1.29 ± 1.45	1.15 ± 0.30	1.50 ± 0.68	1.54 ± 0.86	1.36 ± 0.37
C20:3n-6 (μg/ml)	21.36 ± 10.65	29.78 ± 10.06♱	29.06 ± 17.47	37.00 ± 16.44∗♱	24.06 ± 8.59
C20:4n-6 (μg/ml)	163.21 ± 62.70	132.47 ± 40.96∗	175.06 ± 61.08	151.33 ± 42.28	183.84 ± 47.08
C20:5n-3 (μg/ml)	60.09 ± 44.89∗	86.89 ± 30.13∗♱	40.98 ± 28.97	75.94 ± 30.24∗♱	36.08 ± 22.78
C22:0 (μg/ml)	1.54 ± 0.77∗	1.66 ± 1.06∗	1.46 ± 0.86∗	1.75 ± 0.97∗	0.90 ± 0.42
C22:1n-9 (μg/ml)	2.63 ± 0.98∗	2.62 ± 2.20	2.72 ± 1.21∗	2.99 ± 2.23	1.76 ± 0.65
C22:5n-3 (μg/ml)	17.82 ± 6.60	25.37 ± 6.26∗♱	19.06 ± 5.95∗	25.44 ± 8.23∗♱	15.73 ± 3.24
C22:6n-3 (μg/ml)	98.26 ± 44.55	114.63 ± 44.99♱	96.71 ± 41.14	100.11 ± 38.07	84.92 ± 27.89
C24:0 (μg/ml)	1.46 ± 0.56∗	1.42 ± 0.57∗	1.62 ± 0.65∗	1.58 ± 8.25∗	0.96 ± 0.28
C24:1n-9 (μg/ml)	2.41 ± 1.58	1.90 ± 1.32	2.42 ± 1.59	2.33 ± 8.26	1.60 ± 0.47
C22:4n-6 (μg/ml)	3.86 ± 2.07	3.42 ± 1.19	4.71 ± 1.71∗	4.33 ± 2.07	3.72 ± 0.92
Total (μg/ml)	2515.84 ± 703.48	2401.55 ± 347.34	3096.36 ± 716.48∗	2658.56 ± 8.27	2417.48 ± 363.46

*AN-R* Anorexia Nervosa Restricting type, *AN-BP* Anorexia Nervosa Binge-Eating/Purging type

Values presented as the mean ± SD for the number of observations indicated

Significant differences in the comparisons of the AN-R and AN-BP groups with controls: ∗*P* < 0.05

Significant differences between values on admission and after 3 months: ♱*P* < 0.05

**Fig. 2:**
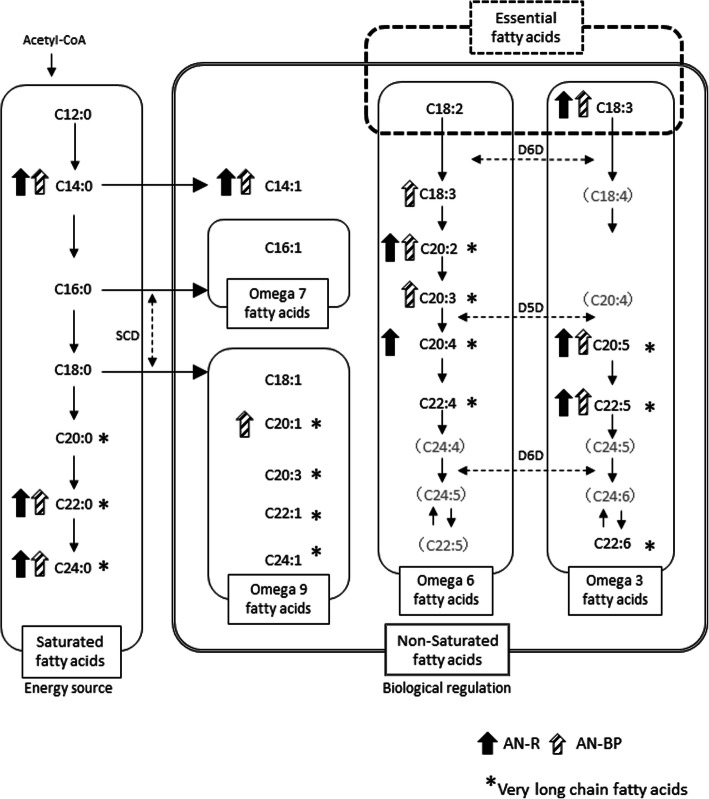
Metabolism of fatty acids in the AN-R and AN-BP groups after 3 months of treatment. AN-R: Anorexia Nervosa restricting type, AN-BP: Anorexia Nervosa Binge-Eating/Purging type, SCD: stearoyl-CoA desaturase, Δ6D: delta-6 desaturase, Δ5D: delta-5 desaturase. The black and the oblique line arrows show the fatty acid levels in the AN-R and AN-BP groups, respectively. The arrows indicate fatty acid levels that are significantly higher in comparison with the healthy controls. Significance level of the difference in comparison with the controls: *P* < 0.05

After 3 months of treatment, the AN-R group had significantly higher levels of 14C or longer fatty acids (saturated fatty acids, MUFAs, and PUFAs compared to those in the control group (Fig. 2). Moreover, after 3 months of treatment, the AN-BP group had significantly higher levels of 14C or longer fatty acids (saturated fatty acids, MUFAs, and PUFAs) compared to those in the control group (Fig. 2). For most parameters, those levels were approximately 1–2 times higher than equivalent values in healthy controls.

Furthermore, we compared the data on admission with that collected 3 months later. Whereas the AN-R group showed a significant increase in the levels of both saturated and unsaturated fatty acids (C14:0, MUFA: C14:1n-5, PUFAs: C20:3n-6, C20:5n-3, C22:5n-3, C22:6n-3), the AN-BP group showed an increase in only the PUFAs (C20:3n-6, C20:5n-3, C22:5n-3). Additionally, the levels of MUFAs (C16:1n-7 and C18:1n-9) decreased significantly in the AN-BP group, such that there was no longer a significant difference between the AN-BP group and the control group.

The lineolic acid (LA) to alpha-lineolic acid (ALA) ratio (C18:2/C18:3: n-6/n-3) on admission was 253.3 ± 188.7 and 169.4 ± 116.2 in patients with AN-R and AN-BP, respectively. The arachidonic acid (ARA) to eicosapentaenoic acid (EPA) ratios (C20:4/C20:5: n-6/n-3) on admission were 5.42 ± 5.8 and 6.07 ± 3.80 in the AN-R and AN-BP groups, respectively; these values were not significantly different from those of the control group (171. ± 95.4 and 6.7 ± 3.7, respectively). The ratios of LA/ALA (C18:2/C18.3: n-6/n-3) 3 months after admission were 191.4 ± 162.3 and 164.4 ± 130.06 in the AN-R and AN-BP groups, respectively. The ratios of ARA/EPA (C20:4/C20:5: n-6/n-3) 3 months after admission were 1.65 ± 0.5 and 2.3 ± 1.1 in the AN-R and AN-BP groups, respectively. There was no statistically significant difference in LA/ALA ratio between the groups. However, the ratio of ARA/EPA 3 months after admission in the AN-R and AN-BP groups were significantly lower than that of controls (*P* < 0.001 and *P* < 0.001, respectively).

In summary, at the time of admission, while the AN-R group had high levels of 18-24C fatty acids, the AN-BP group had high levels of 14-24C fatty acids. After 3 months of treatment, both groups showed high levels of 14-24C fatty acids. The VLCFA (C22:00, C24:0) and LCFA (C18:3) levels in patients with AN were consistently elevated in both the AN-R and AN-BP group relative to control subjects. Following treatment, n-3 fatty acids become more dominant than n-6 fatty acids.

## Discussion

The present study demonstrates an association between different types of abnormal eating behavior and plasma fatty acid profiles in patients with AN. We hypothesized that the lipid metabolism abnormalities seen in undernourished AN patients are influenced by eating behavior and genetic factors. First, on admission, high levels of essential fatty acid (C18:3n-3) and VLCFAs were seen in both the AN-R and AN-BP group, while high levels of LCFAs (saturated fatty acids, MUFAs, and PUFAs) were seen only in the AN-BP group. Essential fatty acids are those that humans cannot synthesize, and therefore, must ingest because the body requires them for good health. In contrast, non-essential fatty acids are synthesized in the body, in particular, saturated fatty acids, which are synthesized via carbohydrate metabolism [23]. A previous study has reported that patients in the AN-BP group often consume high-calorie meals when binge eating and before purging [24]. The study showed high levels of a range of essential and non-essential fatty acids (14-24C) in the AN-BP group. We found that the fatty acid profiles of AN-R patients had changed after 3 months of treatment. It is most interesting that levels of 6 FAs (out of 12 already elevated at admission in AN patients relative to controls) increased further during treatment, with 3 of them being n-3 FAs. These findings suggest that binge eating and feeding with inpatient care promote fatty acid absorption and synthesis. However, the methods used in this study preclude discussions about underlying mechanisms. It takes several years for the improvement of fatty acid metabolism abnormalities for healthy subjects [25]; 3-months of treatment may be insufficient for lipid dysregulation to reverse.

Second, in the AN-R and AN-BP groups, the levels of VLCFA and LCFA (saturated fatty acids, PUFAs, and MUFAs) were higher than in the control group 3 months after hospitalization. In addition, post-treatment levels of VLCFA and some LCFAs (3 out of 6 of these fatty acids were n-3fatty acids) in patients with AN-R were consistently elevated relative to those in control subjects and to those in the AN-R group at admission. Metabolic disorders remained present despite improvement in nutritional status and feeding behavior after 3 months of treatment.

Hunna et al. reported eight genes (CADM1, MGMT, FOXP1, PTBP2, CDH10, NSUN3, NCKIPSD, ASB3, ERLEC1) that may play a role in the pathogenesis of AN [10]. These genes are related to both psychiatric diseases and metabolic parameters associated with low weight [10]. Our study suggests that these genes have some indirect relation to the metabolism of fatty lipids. In addition, the PUFAs derived from meals affect AN risk through the interaction with an AN susceptibility gene [17, 26]. Epoxide hydrolase 2 (EPHX2) influences AN risk through in vivo interaction with dietary PUFAs [19]. These results suggest that genetic factors affect the absorption and synthesis of fatty acids. As shown in this study, AN appears to be associated with VLCFAs (C22:0 and C24:0) and LCFA (C18:3n-3). There are two plausible explanations of this phenomenon. First, genetic changes associated with lipid metabolism abnormality are a genetic predisposition toward AN. Second, absorption and synthesis of fatty acids available in a meal is accelerated in patients as a result of treatment for AN.

In addition, in the presence of chronic malnutrition, patterns of phospholipid fatty acids, and cholesteryl esters indicate changes characteristic of essential fatty acid deficiency of moderate degree [27]. In AN, the n-6/n-3 ratio at admission was not different from that in controls, and it decreased relative to the levels observed in controls after nutrition treatment. The mechanisms of this effect remain unclear. A genetic diathetic mechanism might explain this phenomenon. Given the 3:6 fatty acid ratio, treatment for AN might bring patients closer to the desired body fat composition The impact of genetic variation may be partly to associated with this finding. It is important to note the competition over available enzymes between two classes (n-6 and n-3) of PUFAs. The fatty acids of each class compete for the same desaturase. Under such conditions, if the concentration of n-3 exceeds that of n-6, the inflammatory response might be reduced. In Japan, likely due to high consumption of fish, population levels of EPA and DHA of red blood cells are approximately twice those of the European and Northern American populations. The desired n-6/n-3 ratio is therefore likely lower among the Japanese than among the Western populations. Despite similarities in inpatient meals between Japan, Europe, and Northern America, data comparisons should be performed with caution.

After patients are hospitalized, feeding behavior and malnutrition state change to a more normalized pattern. We estimated that lipid metabolism was similar in the AN-R and AN-BP groups; however, there were between-group differences at 3 months after hospitalization. Steinhauser et al. speculate that acute hypoleptinemia induces a shift from carbohydrate to lipid metabolism during acute starvation in healthy people [25]. Whether leptin becomes the metabolic key in the acute weight recovery phase remains to be elucidated. Further research is required, including an investigation into genetic factors and eating behaviors, and their impact on nutritional recovery from a state where leptin production is close to absent.

Thirdly, beta-oxidation is an important catabolic process by which fatty acids are broken down and converted into acetyl-CoA [28]. Although beta-oxidation of fatty acids usually takes place in the mitochondria, that of VLCFAs occurs selectively in the peroxisomes [28] and requires flavin adenine dinucleotide (FAD) [29, 30]. FT3 is associated with metabolism of FAD [29, 30]; therefore, low FT3 levels play a role in the disruption of FAD metabolism in patients with AN [28]. It has been reported that liver function abnormalities due to acute malnutrition are associated with autophagy in patients with AN [9]. Chronic malnutrition, such as marasmus, has been reported to cause fatty liver due to the accumulation of VLCFAs [18]. Both acute and chronic malnutrition were represented in this study. We speculate that beta-oxidation might be decreased in the peroxisomes of patients with AN, based on the chronic malnutrition state in children. However, because FAD activity was not measured, we could not reach any conclusions regarding its effects on metabolism.

The mechanisms of lipid metabolism abnormalities in individuals with AN remain unclear, and it is not known whether they are due to genetic factors or a protective response to starvation.

This study has a few limitations. First, we did not evaluate the beta-oxidation of VLCFAs in the peroxisomes. Second, we measured only the total level of plasma fatty acids, not the level included in erythrocytes, cholesteryl ester level, and TG level in blood serum. Third, we did not measure the different types of lipoproteins included in the fatty acids. Fourth, the patients studied had been ill for > 5 years. Starvation associated with AN might be a unique metabolic state; however, we were not able to determine this based on our population. Long-term AN results in organ damage; our findings should be verified in a population with a short duration of AN. Finally, no follow-up data were available after the patients left the hospital. Further studies are, therefore, needed to address these limitations. Especially, we would like to follow up study for 2 years post recovery of eating disorders with the same parameters.

## Conclusions

In conclusion, levels of many LCFA types seem to be affected by eating behaviors, and both the AN-R and AN-BP groups had high concentrations of plasma VLCFAs. VLCFA may be an important fatty acid for understanding fat metabolism resulting from genetic factors and the starvation state in AN.

## Data Availability

The datasets used and/or analyzed during the current study are available from the corresponding author on reasonable request.

## Abbreviations

AN: Anorexia nervosa
BMI: Body mass index
VLCFAs: Very long chain fatty acids
AN-R: AN-restricting type
AN-BP: AN-Binge-Eating/Purging type
SCD: Stearoyl-CoA desaturase
D6D: Delta-6 desaturase
D5D: Delta-5 desaturase
SD: Mean ± standard deviation
TG: Triglyceride
FT3: Free triiodothyronine
FT4: Free thyroxine
AST: Aspartate aminotransferase
ALT: Alanine aminotransferase
LCFAs: Long chain fatty acids
FAD: Flavin adenine dinucleotide
