# Correction to: Very long chain fatty acids are an important marker of nutritional status in patients with anorexia nervosa: a case control study

**DOI:** 10.1186/s13030-020-00192-w

**Published:** 2020-08-12

**Authors:** Miki Shimizu, Keisuke Kawai, Makoto Yamashita, Masayasu Shoji, Shu Takakura, Tomokazu Hata, Megumi Nakashima, Keita Tatsushima, Kazunari Tanaka, Nobuyuki Sudo

**Affiliations:** 1 Department of Psychosomatic Medicine, Graduate School of Medical Science, Kyushu University, 3-1-1 Higashi-ku, Fukuoka, 812-8582 Japan; 2 Department of Psychosomatic Medicine, Kohnodai Hospital, National Center for Global Health Medicine, 1-7-1, Kohnodai, Ichikawa City, Chiba 272-8516 Japan; 3 Department of Nutrition, University of Nagasaki, 1-1-1 Manabino, Nagayo-cho, Nishisonogi-gun, Nagasaki, 851-2195 Japan

**Correction to: Biopsychosoc Med (2020) 14:14**

**https://doi.org/10.1186/s13030-020-00186-8**

Following publication of the original article [1], the authors identified an error in Table 1, Table 2 and Table 3. The dagger mark (♱) in the Tables 1, 2, 3 disappears in the pdf version due to typesetting error. The HTML version is correct.

**Table 1:** Demographic data at admission and after 3 months of treatment

	AN-R (*n* = 38)		AN-BP (*n* = 31)		Control group
	on admission	after 3 months	on admission	after 3 months	(*n* = 25)
Age (years)	28.42 ± 12.39	28.42 ± 12.39	32.43 ± 8.19	32.30 ± 8.19	29.33 ± 6.55
Duration of anorexia (days)	2114.08 ± 3255.41	NR	2629.29 ± 2037.48	NR	NR
Height (cm)	154.44 ± 5.39∗	154.77 ± 5.85∗	158.27 ± 3.59	158.47 ± 3.71	160.16 ± 3.02
Body weight (kg)	30.73 ± 5.50∗	37.86 ± 6.04∗†	30.33 ± 3.76∗	38.12 ± 5.03∗†	51.99 ± 4.79
Body mass index (kg/m^2^)	12.81 ± 2.00∗	15.77 ± 2.15∗†	12.17 ± 1.58∗	15.25 ± 2.00∗†	20.20 ± 1.91
Fat mass (g)	3498.3 ± 1611.31	8272.79 ± 3604.42†	4054.34 ± 1572.67	9557.05 ± 5310.85†	NR
Lean body mass (g)	27,283.02 ± 3242.18	30,321.15 ± 3556.30†	26,914.63 ± 2493.5	26,616.08 ± 2334.50†	NR
Percentage of fat mass (%)	10.62 ± 3.81	20.04 ± 7.09†	12.07 ± 3.70	20.42 ± 3.77†	NR

^1^*AN-R* Anorexia Nervosa Restricting type, *AN-BP* Anorexia Nervosa Binge-Eating/Purging type

^2^NR: denotes data not reported. Values presented as the mean ± SD for the number of observations indicated

^3^Significant differences in the comparisons of AN-R and AN-BP with controls: ∗*P* < 0.05

^4^Significant differences between values on admission and after 3 months: †*P* < 0.05

**Table 2:** Biochemical parameters on admission and after 3 months of treatment

	AN-R (*n* = 38)		AN-BP (*n* = 31)		Control group
	on admission	after 3 months	on admission	after 3 months	(*n* = 25)
Glucose (mg/l)	79.79 ± 16.93	80.32 ± 7.73	76.7 ± 12.09∗	81.42 ± 7.42†	85.40 ± 8.95
Total cholesterol (mg/dl)	189.32 ± 60.08	193 ± 37.17	228.83 ± 50.95∗	208.68 ± 42.71	200.16 ± 28.46
Triacylglycerol (mg/dl)	74.24 ± 37.28	73.55 ± 23.06	108.73 ± 47.99∗	83.26 ± 44.51	58.54 ± 19.45
HDL-C (mg/dl)	70.47 ± 19.63	69.91 ± 17.46	74.2 ± 25.32	70.79 ± 15.66	74.24 ± 16.42
TSH (μU/ml)	2.61 ± 2.27	2.31 ± 1.29	1.629 ± 1.16	1.79 ± 0.65	2.01 ± 0.72
FT3 (pg/ml)	1.93 ± 0.60∗	2.71 ± 0.69∗†	2.06 ± 0.53∗	2.35 ± 0.32∗†	3.21 ± 0.40
FT4 (ng/dl)	1.07 ± 0.24∗	0.92 ± 0.18∗†	1.16 ± 0.33	0.92 ± 0.18∗†	1.29 ± 0.20
Cortisol (μg/dl)	20.19 ± 6.56∗	12.79 ± 4.11†	20.33 ± 6.47∗	13.26 ± 4.09†	15.68 ± 3.70
GH (nU/ml)	12.30 ± 19.84∗	3.01 ± 6.78†	8.65 ± 14.00	2.52 ± 1.94†	2.50 ± 2.67
AST (IU/l)	43.82 ± 48.32	34.81 ± 23.30	72.48 ± 203.63	34.83 ± 27.36	NR
ALT (IU/l)	50.34 ± 40.26	47.36 ± 39.67	53.00 ± 99.29	42.17 ± 26.80	NR

^1^*AN-R* Anorexia Nervosa Restricting type, *AN-BP* Anorexia Nervosa Binge-Eating/Purging type

^2^FT3: free triiodothyronine, FT4: free thyroxine, GH: growth hormone

^3^Values presented as the mean ± SD for the number of observations indicated

^4^Significant differences in the comparisons of the AN-R and AN-BP groups with controls: ∗*P* < 0.05

^5^Significant differences between values on admission and after 3 months: †*P* < 0.05

^6^NR: denotes data not reported. The normal ranges for AST and ALT are 7–38 IU/L and 4–44 IU/L, respectively, based on the reference range used by our laboratory

**Table 3:** Fatty acid composition on admission and after 3 months of treatment

	AN-R (*n*=38)		AN-BP (*n*=31)		Control group
	on admission	after 3 months	on admission	after 3 months	(*n* = 25)
C12:0 (μg/ml)	2.62 ± 1.98	3.10 ± 1.41	3.82 ± 3.17	3.84 ± 3.19	3.66 ± 4.16
C14:0 (μg/ml)	16.12 ± 5.59	25.58 ± 7.04∗†	21.78 ± 8.85∗	29.89 ± 13.75∗	15.48 ± 6.20
C14:1n-5 (μg/ml)	1.42 ± 0.71	2.15 ± 0.87∗†	2.27 ± 1.27∗	3.01 ± 1.58∗	1.29 ± 0.65
C16:0 (μg/ml)	488.11 ± 162.94	493.89 ± 93.78	603.93 ± 156.37∗	537.83 ± 130.01	457.96 ± 70.72
C16:1n-7 (μg/ml)	42.55 ± 23.35	46.47 ± 19.09	83.35 ± 44.26∗	55.39 ± 35.42†	35.64 ± 11.76
C18:0 (μg/ml)	231.11 ± 49.62	243.47 ± 39.59	274.06 ± 63.23∗	263.44 ± 41.13	232.80 ± 39.22
C18:1n-9 (μg/ml)	523.11 ± 253.63	406.00 ± 68.07	721.10 ± 209.79∗	458.22 ± 126.84†	461.12 ± 100.02
C18:2n-6 (μg/ml)	794.34 ± 217.25	736.31 ± 170.57	973.61 ± 286.45∗	851.22 ± 173.29	820.48 ± 160.14
C18:3n-6 (μg/ml)	5.71 ± 4.90	8.71 ± 5.44	8.40 ± 5.67	9.94 ± 5.17∗	6.11 ± 3.32
C18:3n-3 (μg/ml)	20.48 ± 8.39∗	22.95 ± 7.01∗	24.86 ± 8.53∗	25.78 ± 10.44∗	15.72 ± 5.57
C20:0 (μg/ml)	2.04 ± 0.57∗	1.83 ± 0.41	2.15 ± 0.66∗	1.82 ± 0.39	1.56 ± 0.29
C20:1n-9 (μg/ml)	6.07 ± 2.40∗	5.54 ± 2.64	5.89 ± 2.19∗	6.57 ± 3.5∗	4.26 ± 0.86
C20:2n-6 (μg/ml)	5.71 ± 3.25	5.83 ± 1.39∗	6.87 ± 3.78∗	7.26 ± 2.01∗	4.69 ± 1.26
C20:3n-9 (μg/ml)	1.29 ± 1.45	1.15 ± 0.30	1.50 ± 0.68	1.54 ± 0.86	1.36 ± 0.37
C20:3n-6 (μg/ml)	21.36 ± 10.65	29.78 ± 10.06†	29.06 ± 17.47	37.00 ± 16.44∗†	24.06 ± 8.59
C20:4n-6 (μg/ml)	163.21 ± 62.70	132.47 ± 40.96∗	175.06 ± 61.08	151.33 ± 42.28	183.84 ± 47.08
C20:5n-3 (μg/ml)	60.09 ± 44.89∗	86.89 ± 30.13∗†	40.98 ± 28.97	75.94 ± 30.24∗†	36.08 ± 22.78
C22:0 (μg/ml)	1.54 ± 0.77∗	1.66 ± 1.06∗	1.46 ± 0.86∗	1.75 ± 0.97∗	0.90 ± 0.42
C22:1n-9 (μg/ml)	2.63 ± 0.98∗	2.62 ± 2.20	2.72 ± 1.21∗	2.99 ± 2.23	1.76 ± 0.65
C22:5n-3 (μg/ml)	17.82 ± 6.60	25.37 ± 6.26∗†	19.06 ± 5.95∗	25.44 ± 8.23∗†	15.73 ± 3.24
C22:6n-3 (μg/ml)	98.26 ± 44.55	114.63 ± 44.99†	96.71 ± 41.14	100.11 ± 38.07	84.92 ± 27.89
C24:0 (μg/ml)	1.46 ± 0.56∗	1.42 ± 0.57∗	1.62 ± 0.65∗	1.58 ± 8.25∗	0.96 ± 0.28
C24:1n-9 (μg/ml)	2.41 ± 1.58	1.90 ± 1.32	2.42 ± 1.59	2.33 ± 8.26	1.60 ± 0.47
C22:4n-6 (μg/ml)	3.86 ± 2.07	3.42 ± 1.19	4.71 ± 1.71∗	4.33 ± 2.07	3.72 ± 0.92
Total (μg/ml)	2515.84 ± 703.48	2401.55 ± 347.34	3096.36 ± 716.48∗	2658.56 ± 8.27	2417.48 ± 363.46

^1^*AN-R* Anorexia Nervosa Restricting type, *AN-BP* Anorexia Nervosa Binge-Eating/Purging type

^2^Values presented as the mean ± SD for the number of observations indicated

^3^Significant differences in the comparisons of the AN-R and AN-BP groups with controls: ∗*P* < 0.05

^4^Significant differences between values on admission and after 3 months: †*P* < 0.05

The correct tables have been included in this correction, and the original article has been corrected.
